# Environmental and sociodemographic risk factors associated with environmentally transmitted zoonoses hospitalisations in Queensland, Australia

**DOI:** 10.1016/j.onehlt.2020.100206

**Published:** 2020-12-17

**Authors:** J. Cortes-Ramirez, D. Vilcins, P. Jagals, R.J. Soares Magalhaes

**Affiliations:** a School of Public Health and Social Work, Queensland University of Technology, Australia; b Children's Health and Environment Program, Child Health Research Centre, The University of Queensland, South Brisbane 4101, Queensland, Australia; c Spatial Epidemiology Laboratory, School of Veterinary Science, The University of Queensland, Gatton, 4343, QLD, Australia

**Keywords:** Emerging infectious diseases, disease mapping decision rules, Bayesian spatial hierarchical model, Integrated nested Laplace approximation, Spatial adjacency matrix, Local government areas

## Abstract

Zoonoses impart a significant public health burden in Australia particularly in Queensland, a state with increasing environmental stress due to extreme weather events and rapid expansion of agriculture and urban developments. Depending on the organism and the environment, a proportion of zoonotic pathogens may survive from hours to years outside the animal host and contaminate the air, water, food, or inanimate objects facilitating their transmission through the environment (i.e. environmentally transmitted). Although most of these zoonotic infections are asymptomatic, severe cases that require hospitalisation are an important indicator of zoonotic infection risk. To date, no studies have investigated the risk of hospitalisation due to environmentally transmitted zoonotic diseases and its association with proxies of sociodemographic and environmental stress. In this study we analysed hospitalisation data for a group of environmentally transmitted zoonoses during a 15-year period using a Bayesian spatial hierarchical model. The analysis incorporated the longest intercensal-year period of consistent Local Government Area (LGA) boundaries in Queensland (1996–2010). Our results showed an increased risk of environmentally transmitted zoonoses hospitalisation in people in occupations such as animal farming, and hunting and trapping animals in natural habitats. This risk was higher in females, compared to the general population. Spatially, the higher risk was in a discrete set of north-eastern, central and southern LGAs of the state, and a probability of 1.5-fold or more risk was identified in two separate LGA clusters in the northeast and south of the state. The increased risk of environmentally transmitted zoonoses hospitalisations in some LGAs indicates that the morbidity due these diseases can be partly attributed to spatial variations in sociodemographic and occupational risk factors in Queensland. The identified high-risk areas can be prioritised for health support and zoonosis control strategies in Queensland.

## Highlights

• Environmentally-transmitted zoonoses hospitalisation are a public health burden.
• Spatial patterns of risk factors associated with hospitalisation risk in Queensland.
• Animal-associated occupations increase the hospitalisation risk for these diseases.
• Higher hospitalisation risk of these diseases in females than the general population.
• Increased risk in north-eastern, central and southern Queensland geographical areas.

## 1 Introduction

More than 60% of human infectious diseases are zoonotic in nature (i.e. transmitted between vertebrate animals and humans) some of which can lead to severe public health emergencies such as the current COVID-19 pandemic [1,2]. Zoonotic pathogens include bacteria, viruses, protozoa, fungi, helminths and arthropods, and their transmission often depends on complex relationships with their hosts and environmental factors [3]. Given the diversity of transmission pathways mediated either by physical environment conditions (including waterborne, airborne, soil-transmitted) and host and vector behaviour (faecal-oral, foodborne, vector-borne), the incidence of infection varies between demographic groups and spatiotemporal scales [4]. The increased morbidity associated with zoonoses represents direct economic and public health impacts across multiple sectors, and integrated multisector approaches for efficient and targeted policy and decision making (i.e. a One Health approach) are required for prevention and control [5].

Global trade, intensification of agricultural practices and human migration have been shown to influence the emergence and spread of zoonotic diseases [6]. The risk of zoonoses increases in areas with agricultural intensification [7] and in animal-associated occupations such as farmers and animal traders, fishermen and hunters and wild life handlers and veterinarians [8,9]. Environments are further stressed by anthropogenic activities such as urbanization and resource/industrial development, which disrupt the ecological balance of habitats and microclimates increasing the risk of emerging infectious diseases [10]. Environmental changes can affect the densities of zoonotic host species composition and vector communities within, and between species populations. This can affect the contact rate between microorganisms and hosts, increasing the exposure to zoonotic pathogens [11].

Zoonotic diseases are an important public health problem in Queensland, the state with the largest number of locally-acquired cases of zoonoses such as Salmonellosis and Campylobacteriosis – the most common foodborne diseases due to zoonotic pathogens in Australia [12]. Queensland has a high incidence of zoonotic vector-borne diseases such as Ross River Virus (RRV) infection and Barmah Forest disease and other zoonotic faecal-oral parasitic diseases including toxocariasis, strongyloidiasis and hookworm infections [[13], [14], [15], [16]]. However, zoonoses with transmission pathways that involve a combination of contaminated environments (e.g. air, soil, water) such as cryptosporidiosis, leptospirosis, melioidosis and Q fever also impart a significant public health burden. For example, Queensland has the highest Q fever incidence in the country, with more than twice the national rate [17]. Zoonoses due to pathogens that survive for long time in soil, waterbodies and air are more difficult to control compared to foodborne, vector-borne or faecal orally transmitted diseases which are typically controlled via manipulating modifiable risk factors. While these environmentally transmitted zoonoses are endemic to some populations in Queensland, little is known about their predominant geographical distributions and the role of sociodemographic and environmental risk factors in their transmission.

Much research into the risk of zoonoses in Queensland has been conducted using animal surveys data or notifications from the National Notifiable Diseases Surveillance System [13,14,18]. Nevertheless, the list of diseases of mandatory notification excludes some zoonoses related to contaminated soil, water and air such as toxoplasmosis, melioidosis and erysipeloid. A more comprehensive analysis of environmentally transmitted zoonoses morbidity can be done using hospitalisations that include notifiable and non-notifiable zoonotic infections coded according to the International Classification of Diseases.

Geo-statistics can be used to analyse data in geographical areas to better understand the environmental determinants of zoonosis morbidity and produce robust measures of disease risk [19]. Spatial epidemiological analyses are increasingly used in studies of notified cases of vector-borne and parasitic zoonoses in Queensland such as RRV and cryptosporidiosis [20,21]. These studies identified geographical clusters of notifications associated with environmental risk factors such as average-maximum temperature and rainfall. However, long-term analyses of hospitalisations due to environmentally transmitted zoonoses have not been done in Queensland. This restricts a comprehensive understanding of health service utilisation of patients with severe clinical presentations and the risk factors associated. This study aims to investigate the role of environmental and sociodemographic factors in the geographical distribution of hospitalisations due to environmentally transmitted zoonoses in Queensland.

## 2 Methods

This is an aggregated time-series analysis of environmentally transmitted zoonoses hospitalisations in Queensland Local Government Areas (LGA) from 1996 to 2010. This is the longest period for which Queensland LGA geographical boundaries are consistent across several census years allowing a 15-year analysis of zoonoses morbidity. Standardised zoonosis hospitalisation rates (zHR) were calculated, per LGA, and a Bayesian spatial hierarchical model measured the association of the zHR with environmental and sociodemographic risk factors. The model used the Integrated Nested Laplace Approximation, an efficient alternative to Markov-Chain-Monte-Carlo methods which are known to be comparatively more computationally intensive [22]. A sensitivity analysis assessed the fit of the model implementing five adjacency matrix specifications (i.e. spatial structure of the LGA-neighbourhood) and three priors. The risk of environmentally transmitted hospitalisation was mapped using the best fit model.

### 2.1 Data sources

Hospitalisation data were obtained from the Queensland Hospital Admitted Patient Data Collection (ethics approval granted by the Children's Health Queensland Hospital and Health Service Human Research Ethics Committee. HREC/16/QRCH/320). These data included diagnoses coded with the International Classification of Diseases (ICD) 10th version (World Health Organization 2010) for records grouped by 5-year age. The zoonoses selected included anthrax, brucellosis, leptospirosis, melioidosis and glanders, Q fever, toxoplasmosis, tularaemia and erysipeloid. The count of each zoonosis hospitalisations and the yearly count per LGA were very small to be representative samples, therefore all combined zoonoses were selected in a single dataset for the 15-year study period.

The residence area of each hospitalisation record was geocoded to a map of LGA-boundaries consistent across the study period produced with the LGA-boundaries in the census years 1996, 2001 and 2006 (appendix). Indirect standardised hospitalisation rate of zoonoses (zHR) were calculated in R using the 2001 Queensland population as the standard (i.e. standard population used by the ABS for demographic statistics) [23].

The census provides data of occupations including farming and fishing, hunting and trapping animals in farms or other natural habitats. These data are provided in a single category according to the Australian and New Zealand Standard Industrial Classification [24]. Indirect standardised rate of people in at-risk occupations were calculated per LGA and the 2001 Index of Socioeconomic Disadvantage (ISD) per LGA [25] were incorporated in the analysis. To adjust for gender differences, indirect standardised female zoonoses hospitalisation rates were calculated per LGA.

Environmental data provided by the Australian Bureau of Meteorology included daily maximum temperature that could be obtained for the period 1999–2010 only, and seasonal rainfall for the period 1996–2010. Mean maximum temperature (in °C) and mean total rainfall (in mm) were calculated per LGA for the study period and scaled x10^−1^ to ensure that the predictor variables appropriately match the zHR.

### 2.2 Analysis

The distribution of the zHR was analysed with a dispersion test in R [26] that identified overdispersion. Additionally, as some LGAs had no zoonoses hospitalisations, zero-inflation was identified with a zero-test [27], therefore a Bayesian Zero-Inflated Negative Binomial regression was implemented using R-INLA [28] to estimate the association of the zHR with the covariates. The linear predictor was defined on the logarithmic scale:

$$\log\left({\mathrm{zHR}}_{i}\right)=\alpha +\beta_{1}X_{1i}+\beta_{2}X_{2i}+\beta_{3}X_{3i}+\beta_{4}X_{4i}+\beta_{5}X_{5i}+\upsilon_{i}+v_{i}$$

where *i* represents the *i*_*th*_ LGA, *α* is the intercept that quantifies the average zHR across all LGAs, *X*_1_ and *X*_2_ are the average-maximum temperature and average rainfall respectively, *X*_3_ is the standardised female zoonoses hospitalisation rate, and *X*_4_, *X*_5_ are the values of the ISD and the standardised rate of people in at-risk occupations. The parameters *υ*_*i*_ and *v*_*i*_ are random effects representing the spatial and non-spatial components in the model. The Besag-York-Mollie specification was used for the structured residual (parameter *υ*_*i*_) [29].

No collinearity was identified for any of the covariates for which a Variance Inflation Factor (VIF) test estimated a *VIF* ≤ 5 [30].

#### 2.2.1 Sensitivity analysis and risk mapping

Bayesian spatial models incorporate priors for the hyperparameters of the spatial and non-spatial components. To identify their effect on the regression estimates, three non-informative priors previously assessed in Bayesian analyses of Queensland geographical areas [31], were compared using the Deviance Information Criterion (DIC) [32]. The effect of five adjacency matrices (i.e. spatial representation of the LGA-neighbourhood) was also compared (appendix).

The specific posterior means (in each LGA) were estimated in the best fit model to map the environmentally transmitted zoonoses hospitalisation risk. Additionally, the probability of the specific posterior mean to be greater than 1.5 (probability of a 1.5 fold higher hospitalisation risk) was calculated from the posterior distribution to be used as a decision rule threshold [33]. Maps were produced with the R-package T-map [34].

## 3 Results

The study cohort consisted of 9192 environmentally transmitted zoonoses hospitalisations across the 15-year period (3396 hospitalisations in females and 5796 in males) (Table 1).

**Table 1:** Environmentally transmitted zoonoses hospitalisations in Queensland (1996–2010).

**Environmentally transmitted zoonosis**	**Number of hospitalisations in the period 1996–2010**
Anthrax	1
Brucellosis	163
Erysipeloid	230
Leptospirosis	1903
Melioidosis and Glanders	431
Q fever	1059
Toxoplasmosis	120
Tularaemia	2

**Fig. 1:**
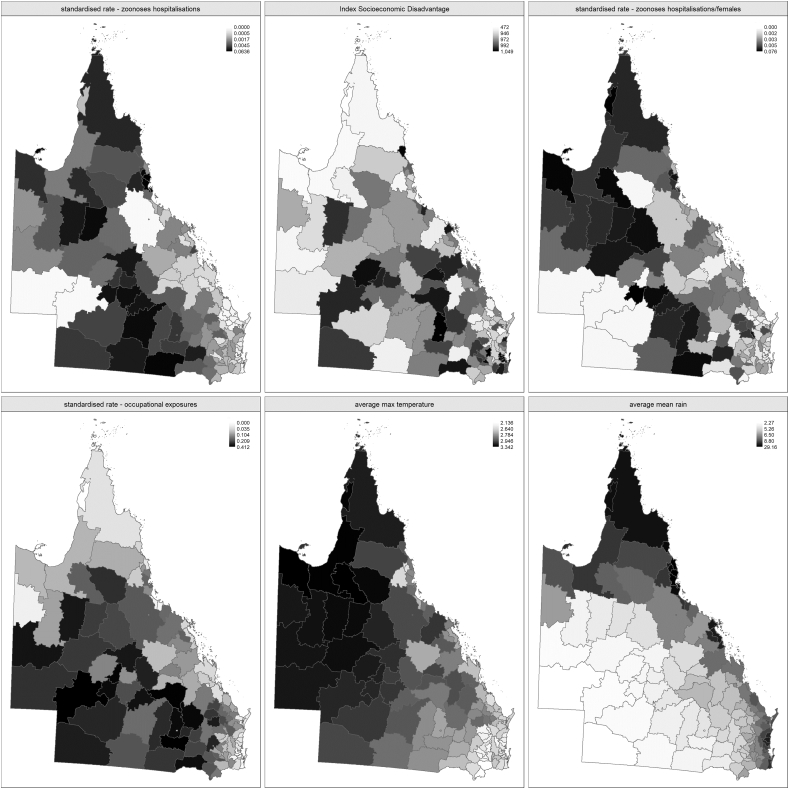
Distribution of zoonoses hospitalisations and socio-environmental covariates in the Queensland LGAs.

Table 2 shows the summary statistics of the zHR and covariates. Fig. 1 shows the geographical distribution of each variable. The highest average maximum temperatures were found in west and north LGAs while there was higher average mean rainfall in northwest, north and all coastal LGAs. Higher hospitalisation rates of people in at-risk occupations were found in south, southwest and central LGAs. The distribution of the zHR and ISD had no evident spatial clusters.

**Table 2:** Descriptive statistics of zoonoses hospitalisations and socio-environmental covariates by LGA in Queensland.

**Variable**	**Mean**	**SD**	**Min**	**Q1**	**Median**	**Q3**	**Max**
Standardised hospitalisation rate of zoonoses (zHR)	0.004	0.008	0.000	0.001	0.002	0.005	0.064
Index of Socioeconomic Disadvantage (ISD)	957.7	69.6	472.1	946.3	972.5	992.4	1048.9
Standardised female zoonoses hospitalisation rate	0.005	0.008	0.000	0.002	0.003	0.005	0.076
Standardised rate of people in at-risk occupations	0.139	0.116	0.000	0.035	0.104	0.211	0.412
Average maximum temperature*	2.809	0.253	2.136	2.640	2.790	2.947	3.342
Average rainfall**	8.037	4.527	2.269	5.264	6.502	8.861	29.159

Notes. Q1: first quartile; Q3: third quartile; SD: standard deviation; * °C x10^−1^; ** mm x10^−1^.

### 3.1 Bayesian spatial analysis of zoonoses hospitalisations risk

All models incorporating a queen-specification adjacency matrix (i.e. all surrounding neighbours of the *i*_*th*_ LGA) had a better fit (lower DIC) (Appendix). Of these, each prior produced models with a positive association of zoonoses hospitalisations with each covariate except the average maximum temperature. In these three models, there were strong associations (credible intervals not crossing 1) of the zHR with the standardised female zoonoses hospitalisation rate and standardised rate of people in at-risk occupations. The proportion of spatial variance indicates that 99%, 86% and 94% of the spatial variability was explained by the structured spatial component in each model, respectively (Appendix). The best fit model (prior 2) estimated a 25.5% and 10% higher risk of hospitalisation due to zoonosis in females and people in at-risk occupations, respectively (Table 3).

**Table 3:** Regression estimates, model using a queen adjacency matrix with prior 2.

	**Posterior mean (CI)**	**SD**
		
Intercept	0.001 (0.001-0.07)	10.637
Index of Socioeconomic Disadvantage	1.001 (0.998-1.004)	1.002
Standardised female zoonoses hospitalisation rate	1.255 (1.154-1.365)	1.044
Standardised rate of people in at-risk occupations	1.10 (1.051-1.151)	1.024
Average maximum temperature	0.701 (0.24-2.042)	1.723
Average rainfall	1.025 (0.973-1.078)	1.026

Spatial variance: 0.86. CI: 95% Credible Interval; SD: Standard Deviation.

### 3.2 Specific LGAs

**Fig. 2:**
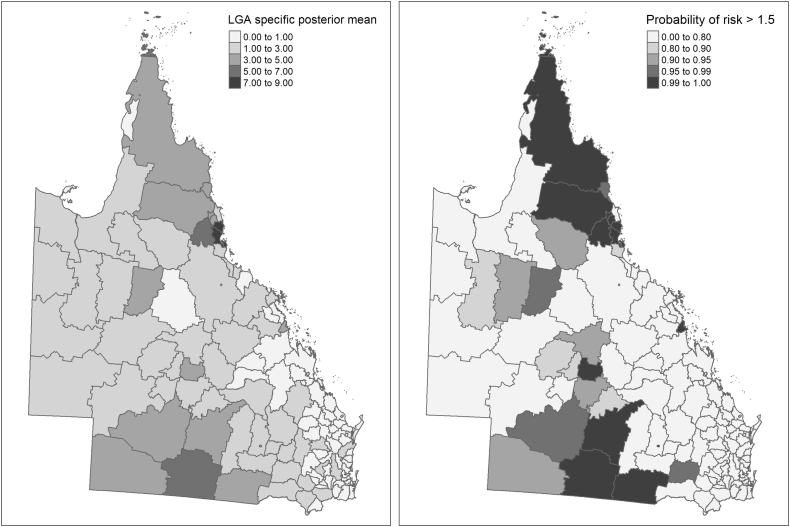
Distribution of the risk and probability of excess of risk of zoonosis hospitalisation in Queensland.

The posterior mean and the probability of excess of risk of environmentally transmitted zoonoses hospitalisation in each LGA are mapped in Fig. 2. A four-fold or greater risk of hospitalisation was found in nine LGAs: Cardwell, Johnstone, Torres, Herberton, Eacham (north and northeast Queensland); Paroo, Murweh, Roma (South Queensland); and Barcaldine (central Queensland). All LGAs with a specific posterior mean ≥ 3 were found in two clusters in northeast and south Queensland with exemption of Sarina (east central-coast), Richmond (central west) and Barcaldine (central Queensland). There was a 99% or higher probability of a 1.5-fold risk of environmentally transmitted zoonoses hospitalisation in 16 LGAs (Herberton, Murweh, Paroo, Roma, Cardwell, Mareeba, Sarina, Atherton, Goondiwindi, Torres, Barcaldine, Eacham, Cook, Balonne, Johnstone and Douglas) (Supplementary material, and specific risk and probability of excess risk data) [49]

## 4 Discussion

We estimated the association between hospitalisations due to environmentally transmitted zoonoses and environmental and sociodemographic risk factors in the Queensland Local Government Areas (LGAs) and mapped the higher risk areas. The analysis used a comprehensive state-wide dataset to provide reliable information on the geographical distribution of hospitalisations in Queensland and implemented a robust spatial modelling framework that accounts for geographical interdependencies between geographical areas. Our approach uncovered associations between environmentally transmitted zoonoses hospitalisations and environmental and sociodemographic risk factors (especially, gender and occupations at risk). It also enabled quantification of geographical variation of hospitalisation risk for specific LGAs. This is the first study to map the risk of environmentally transmitted zoonoses hospitalisations in Queensland.

Occupation is an important consideration regarding zoonotic disease risk because of the potential for people in certain animal-associated occupations to be exposed to infected animals and their environments [35]. We found that people engaged in animal-associated occupations have 10% higher risk of environmentally transmitted zoonosis hospitalisation in Queensland. Previous research on hospitalisations due to zoonoses in Queensland found that 42% of patients hospitalised due to tick typhus (i.e. *Rickettsia australis*) infection had hobbies and or occupations such as bushwalking/orienteering, botanist, wildlife ranger and farm worker/grazier [36]. The increased hospitalisation risk in animal-associated occupations found in this study concur with the findings of previous research to consider occupations at risk as significant predictors of severe zoonoses in Queensland. These exposures characterise the risk posed by the emerging abiotic stress in environments affected by demographic and development pressures [10]. The occupations at risk identified in this study are associated with commercial activities related to environmental interventions such as farming, hunting and trapping animals which can disrupt the ecological balance of habitats and pose a risk of exposure to zoonotic pathogens that survive for long periods in soil, water and air.

Our findings suggest that gender differences can play an important role in the severity of these infections in that we found a higher risk of environmentally transmitted zoonosis hospitalisation in females in Queensland. The disproportional risk of hospitalisation in women could be associated with the presence of perinatal comorbidities. Some environmentally transmitted zoonotic infections can cause abortion and determine foetal development anomalies, thus increasing the risk of hospitalisation during pregnancy and the perinatal period [37,38]. A higher prevalence of cryptosporidiosis and H5N1 virus infection has been identified in women in certain age groups in relationship with socioeconomic and occupational roles such as farming, feeding, purchasing, and handling sick poultry [38,39]. Other studies have found higher risk of hospitalisation due to infection of potential zoonotic pathogens (*Clostridium difficile*) in women over 45 years [40]. As our study estimated age-adjusted hospitalisation rates (all age groups) we did not identify hospitalisation risk related to any specific age group. Beyond the increased risk of perinatal and congenital morbidity and occupational exposures, there is little research on gender factors linked to zoonoses. Experimental studies in animals have found sex differences in the immune response to infectious inflammatory diseases most likely as a consequence of excessive and damaging inflammatory response though these mechanisms have not been assessed in humans [41]. Further research that considers specific age groups and individual risk factors in both sexes is needed to explore and uncover disease specific gender patterns in zoonotic diseases.

In this study we set a threshold of 1.5-fold risk of hospitalisation and in doing so we have identified Queensland LGAs with the highest probability (>98%) to surpass this excess of risk to assist decision making [42]. Disease mapping decision rules have previously been reported to support surveillance programs and design health rankings linked to public health strategies [43]. Our results demonstrate that severe morbidity due to environmentally transmitted zoonoses is localised to a discrete set of Queensland LGAs. We identified a clustering trend of higher risk of environmentally transmitted zoonoses hospitalisation in northeast and south-central Queensland LGAs. Most high-risk LGAs identified in this study are remote locations which highlight important challenges in terms of health care access and utilisation. Populations in Australian remote areas have poorer access to primary health care compared to urban areas, which can lead to inadequate access to medical treatment of severe diseases [44]. The specific LGAs of higher risk of environmentally transmitted zoonoses identified in this study suggests areas in more need of access to health care support to mitigate the health effects of these infections.

We used a hospitalisation data analysis, which selects severe cases of zoonoses and provides key information on their burden of disease and associated public health costs. Furthermore, the higher risk estimates of environmentally transmitted zoonoses in north-eastern LGAs in this study correspond with previous spatial analyses of zoonotic infections utilising notification data in Queensland. A higher risk of RRV transmission has been identified in north-eastern LGAs while clusters of increased risk of cryptosporidiosis have been found in north, central, and south Queensland LGAs after adjusting for socioeconomic disadvantage and temperature [20,21]. We focused on hospitalisation data for a selected group of zoonoses many of which have several transmission pathways that related to contaminated environmental media such as water, food and or soil. Further studies can be conducted in the hot-spots identified in our results.

### 4.1 Limitations

The zoonoses hospitalisation data were made available as aggregated number of cases by age-group, per year, therefore there is a risk of ecological bias (i.e. aggregated data analysis where confounding factors at the group level can produce spurious associations). In addition, regression-dilution bias could be introduced in the independent variables for which data were also aggregated at the LGA level, which could partially explain the very small effect sizes of the covariates. To reduce the risk of bias introduced by the analysis of aggregated data, we adjusted hospitalisations per age-group to control the effect of structural variations between the age groups. We also included variables expressed as rates calculated with the same standardisation method than the dependent variable, rather than crude rates or percentages as predictors in the models. The adjustment of the dependent variable and the covariates using a similar standardisation has been found to reduce the risk of ecological bias [45]. Due to the small numbers of environmentally transmitted hospitalisations in many of the LGAs we did not analyse the data accounting for year variations which would allow to identify temporal trends of zoonoses hospitalisation. We opted for an aggregated time series analysis to have a more representative number of hospitalisations in each geographical area.

## 5 Conclusions

The risk of hospitalisation due to environmentally transmitted zoonoses in Queensland is associated with the geographical distribution of sociodemographic risk factors, especially gender and occupation. This analysis of hospitalisations provides a comprehensive picture of the severity of environmentally transmitted zoonoses and health care service utilisation for these diseases in Queensland. The increased risk of people in animal-associated occupations such as farming, highlights the potentiality of emerging infectious diseases in environments stressed by development activities. Whereas the methods and findings of this study can be used to support decision-making in public and environmental health, further research is needed to understand the causality of higher risk of environmentally transmitted zoonoses in females and people in at-risk occupations.

## Appendix

### A.1 LGA-map

The LGA boundaries in the census years 1996, 2001 and 2006 were assessed to produce a map with LGA boundaries consistent across the whole study period 1996–2010. There were some differences between the LGA boundaries in the census years 2001 and 2006, for which some 2006-LGA were collapsed to overlap the boundaries of a larger 2001-LGA. There were only 2 non-overlapping LGA between 2001 and 2006 (Injinoo and Pormpuraaw) equivalent to 0.021% and 0.031% of the 2006 Queensland population, respectively. These were collapsed into the contiguous 2001-LGA that contained most of their territory (Torres and Cook respectively).

### A.2 Sensitivity analysis

The following priors, previously incorporated in Bayesian analyses for Queensland geographical areas [46], were used to compare the better of fit of the Bayesian spatial models:

$$\mathrm{Prior}\;1.\log\tau_{v}\sim \log\mathrm{Gamma}\;\left(0.50.001\right),\log\tau_{\upsilon }\sim \log\mathrm{Gamma}\;\left(0.50.001\right)$$

$$\mathrm{Prior}\;2.\;\log\tau_{v}\sim \log\mathrm{Gamma}\;\left(0.10.1\right),\log\tau_{\upsilon }\sim \log\mathrm{Gamma}\;\left(0.0010.001\right)$$

$$\mathrm{Prior}\;3.\;\log\tau_{v}\sim \log\mathrm{Gamma}\;\left(0.10.01\right),\log\tau_{\upsilon }\sim \log\mathrm{Gamma}\;\left(0.10.01\right)$$

Five adjacency matrices were also incorporated for comparison. An adjacency matrix was set with a queen-specification (i.e. all surrounding neighbours as in a chess game) [19]. Alternative adjacency matrices were set using a K nearest neighbour algorithm where a “K” number of neighbours is assigned to each geographical area [47]. The K values 5, 7, 9, and 11 were used considering; the average of neighbours of each LGA (K = 5) and previous guidelines that suggest; the square root of the total areas (K = 11), and small K values [19,48]. The distance between the LGAs was calculated using population-weighted centroids generated from the coordinates and populations of gazetted localities (i.e. suburbs and towns). Table A1 shows the DIC of models using each of the priors and the adjacency matrices.

**Table A1:** Deviance Information Criterion of the models compared.

	**Prior 1**	**Prior 2**	**Prior 3**
Bayesian spatial model using a queen AM	831.419	812.9744	818.0272
Bayesian spatial model using a KNN = 5 AM	1286.908	1286.798	1287.077
Bayesian spatial model using a KNN = 7 AM	1286.971	1286.814	1287.075
Bayesian spatial model using a KNN = 9 AM	1286.966	1286.823	1286.714
Bayesian spatial model using a KNN = 11 AM	1286.923	1286.956	1286.730

Notes. AM: Adjacency matrix.

### A.3 Bayesian regression models that incorporated a queen-specification adjacency matrix. Table A2 shows the regression estimates of models using a queen adjacency matrix with prior 1,2 and 3.

**Table A2:** Summary of the Bayesian spatial regression models using each prior.

	**Posterior mean (CI)**	**SD**	**DIC**	**Spatial variance**
**Model prior 1**				
Intercept	0.001 (0.001–0.084)	10.273	831.42	0.99
Index of Socioeconomic Disadvantage	1.001 (0.997–1.004)	1.002		
Standardised female zoonoses hospitalisation rate	1.248 (1.151–1.353)	1.042		
Standardised rate of people in at-risk occupations	1.093 (1.046–1.143)	1.023		
Average maximum temperature	0.716 (0.247–2.075)	1.718		
Average rainfall	1.017 (0.965–1.07)	1.027		

**Model prior 2**				
Intercept	0.001 (0.001–0.07)	10.637	812.97	0.86
Index of Socioeconomic Disadvantage	1.001 (0.998–1.004)	1.002		
Standardised female zoonoses hospitalisation rate	1.255 (1.154–1.365)	1.044		
Standardised rate of people in at-risk occupations	1.10 (1.051–1.151)	1.024		
Average maximum temperature	0.701 (0.24–2.042)	1.723		
Average rainfall	1.025 (0.973–1.078)	1.026		

**Model prior 3**				
Intercept	0.001 (0.001–0.08)	10.553	818.03	0.94
Index of Socioeconomic Disadvantage	1.001 (0.997–1.004)	1.002		
Standardised female zoonoses hospitalisation rate	1.25 (1.152–1.357)	1.043		
Standardised rate of people in at-risk occupations	1.097 (1.048–1.147)	1.023		
Average maximum temperature	0.705 (0.241–2.062)	1.725		
Average rainfall	1.02 (0.969–1.074)	1.026		

Notes. CI: 95% Credible Interval; SD: Standard Deviation; DIC: Deviation Information Criterion.

#### Author statement

Javier Cortes-Ramirez: conceptualisation, methodology, data collection and analysis, writing -original draft preparation.

Dwan Vilcins: data collection, writing -reviewing and editing.

Paul Jagals: writing -reviewing and editing.

Ricardo J. Soares Magalhaes: analysis, writing -reviewing and editing.

## Declaration of Competing Interest

The authors declare that they have no known competing financial interests or personal relationships that could have appeared to influence the work reported in this paper.

## Appendix A Supplementary data

Supplementary material 1 Image 1

Supplementary material 2 Image 2
